# E2F2 and TFDP1 as novel systemic lupus erythematosus (SLE) diagnostic markers and therapeutic targets based on machine learning and experimental study

**DOI:** 10.1186/s41065-026-00687-6

**Published:** 2026-05-11

**Authors:** Qingbo Pan, Xuliang Yu, Jin Zhu, Xiaojin Zheng

**Affiliations:** Department of Clinical Laboratory, The Quzhou Affiliated Hospital of Wenzhou Medical University (Quzhou People’s Hospital), No. 100, Minjiang Avenue, Quzhou, 324000 Zhejiang China

**Keywords:** SLE, E2F2, TFDP1, Machine learning, Diagnosis

## Abstract

**Background:**

Systemic lupus erythematosus (SLE) is a chronic autoimmune disease influenced by multiple genetic and environmental factors.. This study used bioinformatics to identify new diagnostic biomarkers and explore the pathogenesis of SLE.

**Method:**

Three array datasets of peripheral blood mononuclear cells (PBMCs) from SLE patients and control subjects were obtained from GEO, including GSE82221 and GSE11909 (merged for feature discovery) and GSE154851 (independent validation/machine learning training dataset). RRA analysis was used to identify statistically changed DEGs. GO and KEGG analyses explored biological mechanisms. Consistent clustering and machine learning models were employed to identify diagnostic biomarkers, further assessed using ROC and DCA analysis. The proportions of 22 immune cells in SLE patients were calculated via CIBERSORT algorithm, and correlations between biomarkers and immune cells were explored. Data from 76 clinical blood samples were collected, including ALT, AST, CR, BUN, CRP, PLT, WBC, lymphocyte (%), and neutrophil (%). RT-qPCR was used to validate the expression of diagnostic biomarkers.

**Results:**

A total of 62 DEGs were identified, mainly involved in the cell cycle and TGF-beta signaling pathway. Six candidate diagnostic biomarkers for SLE were found: E2F2, KIAA0319L, TRIM58, MMP8, FKBP5, and TFDP1. E2F2 and TFDP1 showed AUC values > 0.7 in training and validation datasets. CIBERSORT analysis revealed disturbed immune cell types in SLE patients, with plasma cells most relevant to E2F2 and TFDP1 expression. Expression of E2F2 and TFDP1 was upregulated in patient samples. The diagnostic models, including E2F2 and TFDP1, performed better than those without these biomarkers.

**Conclusion:**

Our study identifies E2F2 and TFDP1 as potential diagnostic biomarkers for SLE patients and uncovers their most relevant immune cells.

## Introduction

Systemic lupus erythematosus (SLE) is a multifactorial, systemic autoimmune disease that impacts various organs and systems, including the skin, joints, heart, kidneys, blood, and nervous system [1]. In SLE patients, the immune system erroneously targets healthy cells and tissues, usually resulting in a range of pathological manifestations like rashes, fever, and anemia. In some severe cases, SLE can induce significant complications such as lupus nephritis, multiple organ failure, autoimmune erythropenia, and neurological disorders [2]. While patients with mild SLE may experience chronic debilitating health issues, and the condition may become life-threatening when major organs are involved. It is estimated that the global prevalence of SLE ranges from 30 to 50 per 100,000 individuals; notably, 90% of the affected patients are female, with the peak age of onset occurring between late adolescence and early adulthood (teens to early 40 s) [3]. Currently, the number of SLE patients keeps increasing worldwide. As suggested by the preliminary epidemiological data from China, there are approximately 860 thousand to 1.3 million individuals living with SLE in the country.

The clinical manifestations of SLE are highly complex and prone to relapse following periods of remission; moreover, the course of disease tends to be prolonged and recurrent, seriously jeopardizing human health [4]. Clinical studies have demonstrated that early diagnosis and appropriate treatment can effectively halt or delay disease progression while preventing irreversible tissue and organ damage [5], thus significantly enhancing the quality of life for those afflicted by SLE.

Current diagnostic biomarkers for SLE—including antinuclear antibodies (ANA), complement proteins (C3/C4), and anti-dsDNA antibodies—face significant limitations. Although ANA exhibits high sensitivity (> 95%), its specificity is suboptimal (10—15% false positives in healthy populations) [6]. Complement levels (C3/C4) fluctuate dynamically and lack consistency during disease monitoring [7], while anti-dsDNA antibodies are present in only 60—70% of SLE patients, limiting their diagnostic coverage. Furthermore, longitudinal studies indicate poor patient compliance with serial serological testing due to invasive sampling and high costs [4, 6, 7]. These limitations underscore an unmet need for non-invasive, stable, and highly specific biomarkers capable of early detection and real-time monitoring of SLE activity. Consequently, there is a critical demand for identifying the highly sensitive, specific, reliable, and non-invasive biomarkers, so as to facilitate early diagnosis and treatment of this complex autoimmune disorder. In recent years, machine learning-based algorithms have been increasingly applied to biological data for biomarker discovery and disease classification across various medical fields [8, 9]. In this study, we leveraged bioinformatics and machine learning approaches to identify novel transcriptional biomarkers for SLE diagnosis.

## Material and method

### Data download, gene expression normalization, and DEGs identification

Three SLE PBMC expression profile datasets (GSE82221, GSE11909, and GSE154851) were obtained from the GEO database (GSE82221: GPL10558/Illumina HumanHT-12 V4.0; GSE11909: GPL96 and GPL97/Affymetrix Human Genome U133A and U133B; GSE154851: GPL10558/Illumina HumanHT-12 V4.0) for comprehensive analysis. The sample distribution across datasets comprised 15 SLE and 25 control specimens in GSE82221, 156 SLE versus 19 controls in GSE11909, and 38 SLE compared with 32 controls in GSE154851. All microarray data underwent standardized preprocessing through R programming, with subsequent calculation of fold change values between patient and control groups. Differential gene expression analysis was executed utilizing the "limma" package in R, implementing a statistical significance threshold of *p* < 0.05 for initial DEG identification, a widely adopted threshold in transcriptomic studies that balances sensitivity and specificity for exploratory biomarker discovery, with subsequent multi-stage filtering to ensure robustness. To enhance analytical rigor, Robust Rank Aggregation (RRA) methodology was applied for consensus DEG detection, employing selection criteria including absolute logarithmic fold change (log|FC|) exceeding 0.2 to ensure biological relevance. This relatively lenient fold-change threshold was chosen to capture modest but biologically meaningful expression changes characteristic of heterogeneous autoimmune conditions such as SLE, where subtle transcriptomic shifts across immune cell subsets can be pathologically relevant [7].

To integrate the GSE82221 and GSE11909 datasets, the ComBat algorithm (R "sva" package) was applied for batch correction to address platform-specific technical variation. The effectiveness of batch correction was validated through principal component analysis (PCA) and boxplot visualization, confirming adequate removal of batch effects (Fig. 2A-B). Subsequent differential expression analysis on the merged dataset was performed using the "limma" package with empirical Bayes moderation, applying thresholds of |log₂FC|> 0.5 and *p* < 0.05 to identify high-confidence DEGs. Potential confounders such as age, sex, and ethnicity were not included as covariates because these clinical variables were inconsistently annotated across the publicly available GEO datasets, precluding reliable covariate adjustment.

### Venn analysis

Interdisciplinary genomic analysis was conducted using the Venn diagram tool (Venn v3.1) from the Bioinformatics & Evolutionary Genomics platform (http://bioinformatics.psb.ugent.be/webtools/Venn/) to identify consensus differentially expressed genes between the Robust Rank Aggregation (RRA) analysis results and integrated multi-dataset findings.

### Biological function and pathway enrichment analyses

Functional annotation integration was conducted through the R-based "ClusterProfiler" package, incorporating comprehensive analyses across Gene Ontology (GO) categories (biological processes, cellular components, molecular functions) and Kyoto Encyclopedia of Genes and Genomes (KEGG) pathway enrichment investigations [10, 11]. Reference pathway datasets were established using the Reactome gene set (c2.cp.reactome.v7.0.symbols.gmt) sourced from the Molecular Signatures Database (MsigDB). Statistical significance in pathway enrichment outcomes was evaluated through false discovery rate (FDR) correction, with an FDR-adjusted *p*-value threshold < 0.05 serving as the criterion for biological pathway prioritization.

### Cluster analysis of the merged dataset

Consensus clustering was implemented via the Sangerbox online platform (http://sangerbox.com/) using the ConsensusClusterPlus algorithm on the integrated dataset. Algorithm stability assessment through cumulative distribution function (CDF) analysis identified the optimal cluster number (K = 2). Concurrently, principal component analysis (PCA) evaluated the discriminative capacity of transcriptional profiles across molecular subgroups.

### Machine learning algorithms

A multimodal diagnostic framework was developed through systematic evaluation of 12 machine learning architectures: Lasso regression, Ridge regression, Stepwise Generalized Linear Model (Stepglm), eXtreme Gradient Boosting (XGBoost), Random Forest (RF), Elastic Net (Enet), Partial Least Squares-GLM (plsRglm), Gradient Boosting Machine (GBM), Naïve Bayes, Linear Discriminant Analysis (LDA), GLM Boosting (glmBoost), and Support Vector Machine (SVM). A total of 113 algorithm combinations (single algorithms and pairwise combinations) were systematically evaluated using the Shengxin Douyacai online platform (http://www.binbiotools.com/), which provides standardized machine learning pipelines with default hyperparameters. The 19 candidate genes identified from consensus clustering of the merged dataset (GSE82221/GSE11909) served as input features. Model training and 10-fold cross-validation were performed on the GSE154851 dataset (38 SLE, 32 controls) as an independent training cohort. External validation was conducted on the merged meta-analysis cohort (GSE82221/GSE11909; 171 SLE, 44 controls), with five key metrics:​​

1. Discriminatory capacity: Area under the receiver operating characteristic curve (AUC)
2. Classification accuracy: (TP + TN)/(TP + TN + FP + FN)
3. Clinical sensitivity: TP/(TP + FN)
4. Clinical specificity: TN/(TN + FP)
5. F1-score: 2 × (Precision × Recall)/(Precision + Recall).

The Random Forest (RF) algorithm, which emerged as the optimal model, was implemented via the online platform using default parameters (500 trees, mtry = √p). This approach ensured reproducibility while avoiding manual hyperparameter tuning bias..

### Identification of the diagnostic markers’ and models’ diagnostic efficacy

A dual-parameter optimization framework was implemented to assess predictive biomarkers, combining receiver operating characteristic (ROC) analysis for discriminatory capacity evaluation and decision curve analysis (DCA) for clinical utility quantification. Computational validation was performed through:1)ROC profiling using the "pROC" R package to determine biomarker classification efficiency; 2) Three-model comparative DCA (performed using the Extreme Smart Analysis online platform, https://www.xsmartanalysis.com/) calculating net benefit differentials against extreme clinical scenarios (full-intervention vs. non-intervention thresholds; 3) Algorithm optimization prioritizing simultaneous maximization of area under the curve (AUC) and clinical decision impact metrics.

### Evaluation of infiltrating immune cells

Cellular heterogeneity was deconvolved using CIBERSORT algorithm (v1.06) with the LM22 leukocyte signature matrix (22 immune cell types) to quantify immune cell proportions from bulk transcriptomic data, employing 1,000 permutation tests (*p* < 0.05 threshold) to assess deconvolution significance. Additionally, xCell (v1.0) was used as a complementary approach for cross-validation of immune cell enrichment estimates.

### Clinical sample collection

This investigation prospectively enrolled 76 hospitalized patients from Quzhou Affiliated Hospital of Wenzhou Medical University (Quzhou People's Hospital) between November 2024 and February 2025 following informed consent acquisition. SLE patients were diagnosed according to the 2019 European League Against Rheumatism (EULAR)/American College of Rheumatology (ACR) classification criteria for SLE [12]. The control group (*n* = 38) consisted of age- and sex-matched hospitalized patients without autoimmune diseases (including orthopedic, cholelithiasis, and other non-autoimmune conditions). Retrospective data extraction from medical records captured comprehensive clinical and laboratory parameters for all participants, with explicit participant agreement secured prior to inclusion. The research protocol received formal ethical authorization from Quzhou People's Hospital's Ethics Review Committee (No. 2025—013), ensuring strict compliance with national regulatory standards and institutional guidelines. In alignment with the study's retrospective design, the Institutional Review Board granted a waiver for individual consent requirements. Biological specimens comprised 76 peripheral blood samples systematically categorized into non-SLE controls (*n* = 38) and SLE cases (*n* = 38).

### RT-qPCR analysis of the mRNA level of E2F2 and TFDP1

Peripheral blood specimens (3 mL) collected in EDTA anticoagulant tubes underwent standardized processing through sequential centrifugation and washing protocols. Following initial mixing with an equal volume of D-Hank's balanced salt solution, samples underwent refrigerated centrifugation (4 °C, 3000 rpm, 10 min) to separate cellular components. The supernatant was carefully decanted, with subsequent resuspension of pelleted cells in fresh D-Hank's solution and repeated centrifugation cycles (2—3 repetitions) to optimize leukocyte isolation. The buffy coat containing mononuclear cells was subsequently aspirated for downstream nucleic acid isolation. RNA isolation employed TRIzol reagent (Invitrogen, USA) followed by reverse transcription using a First Strand cDNA synthesis kit (TaKaRa, Japan). RT-qPCR analyses were conducted on a CFX96 Touch Real-Time PCR platform (Bio-Rad, USA) utilizing gene-specific primers and SYBR Green-based Master Mix (ThermoFisher). Amplification parameters consisted of initial denaturation (95 °C, 10 min), followed by 40 cycles of two-step amplification (95 °C denaturation for 15 s, 60 °C annealing/extension for 1 min), with GAPDH serving as the endogenous normalization control throughout all experimental replicates. Each sample was assayed in triplicate, and no-template controls were included in each run. The 2-ΔΔCt method was utilized to estimate the relative mRNA expression levels of the target genes normalized to GAPDH. The sequences of the primers used in this study were as follows: E2F2, forward 5’−3’-CGGCGCATCTATGACATCACC, and reverse 5’−3’-CATTCCCCTGCCTACCCACTG; TFDP1, forward 5’−3’-ACCACATCTTACCAAACGAGT, and reverse 5’−3’-TCCTCTGTCTTTCCACCTCTA; GAPDH, forward 5’−3’-GGA GCG AGA TCC CTC CAA AAT, and reverse 5’−3’-GGC TGT TGT CAT ACT TCT CAT GG.

### Identification of the most important variables

Predictor importance was assessed through integrative machine learning evaluations employing three distinct algorithms: Least Absolute Shrinkage and Selection Operator (LASSO) regression, Random Forest ensemble modeling, and eXtreme Gradient Boosting (XGBoost). Consensus-based selection criteria identified the most biologically significant biomarkers through intersecting top-ranked features across all algorithmic approaches.

### Statistical analysis

Differential expression patterns of E2F2 and TFDP1 between comparative groups were evaluated using Student's t-test. Statistical computations were implemented through SPSS v23.0 and R programming environment, with statistical significance defined at *P* < 0.05.

## Results

### Identification of key DEGs

To systematically identify robust differential expressed genes (DEGs) in systemic lupus erythematosus (SLE), we adopted a tiered analytical approach combining dataset-specific detection and cross-validation strategies.

**Fig. 1:**
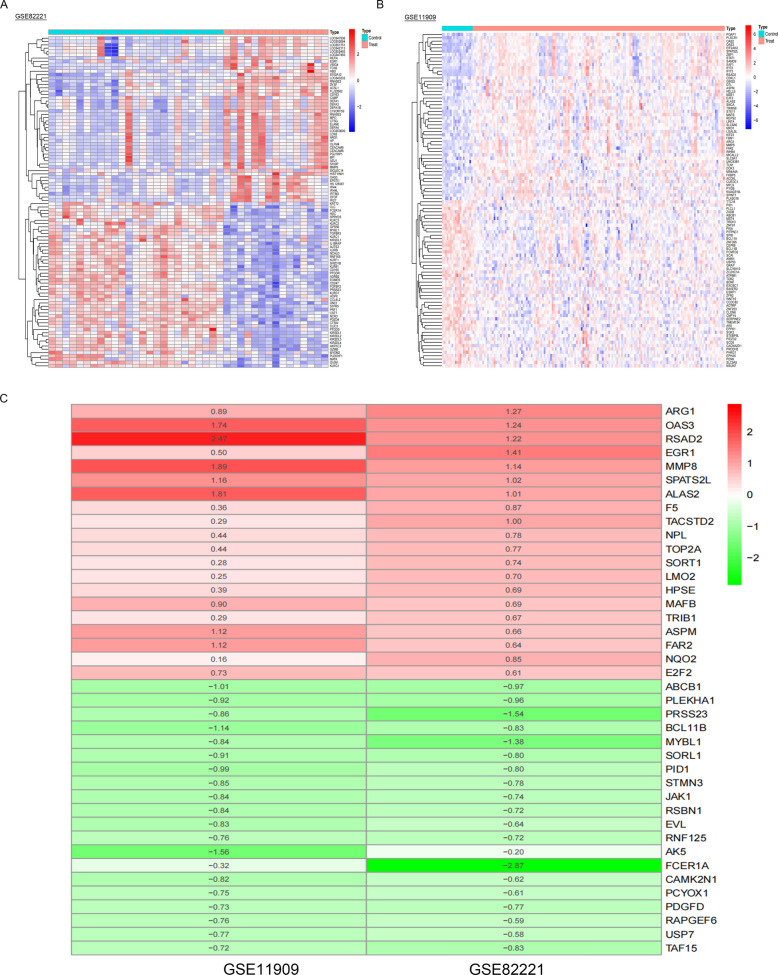
Identification of DEGs. **A** A Heatmap demonstrated the 2371 DEGs in the GSE82221 dataset. **B** A Heatmap demonstrated the 1193 DEGs in the GSE11909 datasets. **C** A Heatmap demonstrated the 1532 robust DEGs between the SLE and healthy. Twenty genes were promoted and twenty genes were suppressed in SLE. Red shows upregulated gene expression and green shows downregulated gene expression in SLE. Numbers in the heatmap indicate log2 FC of genes in the two datasets compared with healthy groups

Initial screening of individual GEO datasets (GSE82221: 15 patients vs. 25 controls; GSE11909: 156 patients vs. 19 controls) using moderated t-test with a significance threshold of *p* < 0.05 and |log_2_FC|> 0.2 threshold revealed 2,371 DEGs (1,164↑/1,207↓) in GSE82221 and 1,193 DEGs (579↑/614↓) in GSE11909. The top 50 DEGs ranked by significance from each dataset were visualized in Fig. 1A and B.

**Fig. 2:**
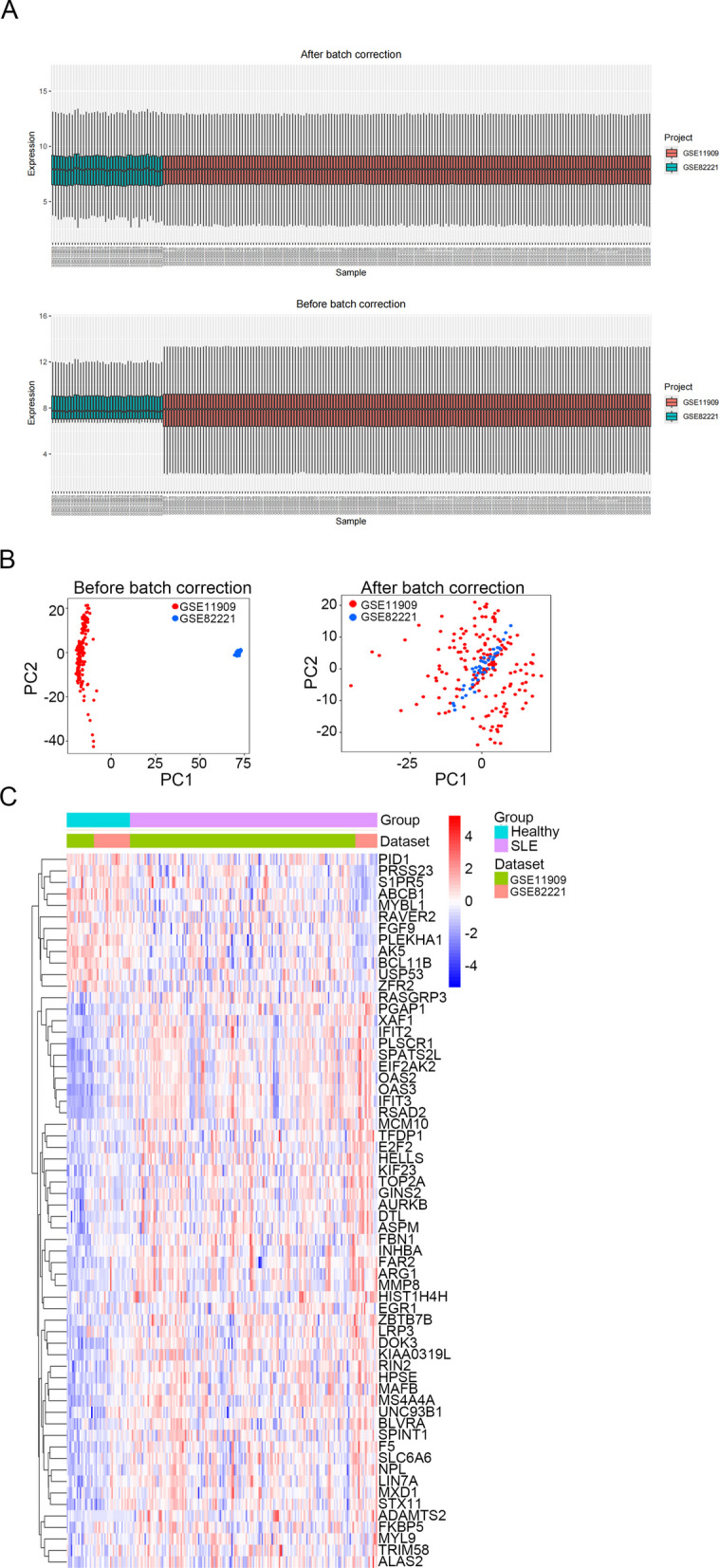
Screening of DEGs for the merged dataset. **A** Normalization of two data sets. **B** Merging of two data sets. **C** A Heatmap demonstrated the 62 DEGs in the merged dataset

To enhance reproducibility across heterogeneous cohorts, we implemented Robust Rank Aggregation (RRA) analysis on both datasets. Applying a |log_2_FC|> 0.2 threshold identified 1,532 concordant DEGs (780↑/752↓), with the top 20 fold-change genes shown in Fig. 1C. Subsequent dataset merging and batch-effect correction (Fig. 2A-B) followed by more stringent filtering (|log_2_FC|> 0.5, *p* < 0.05) yielded 62 high-confidence DEGs (50↑/12↓) (Fig. 2C).

**Fig. 3:**
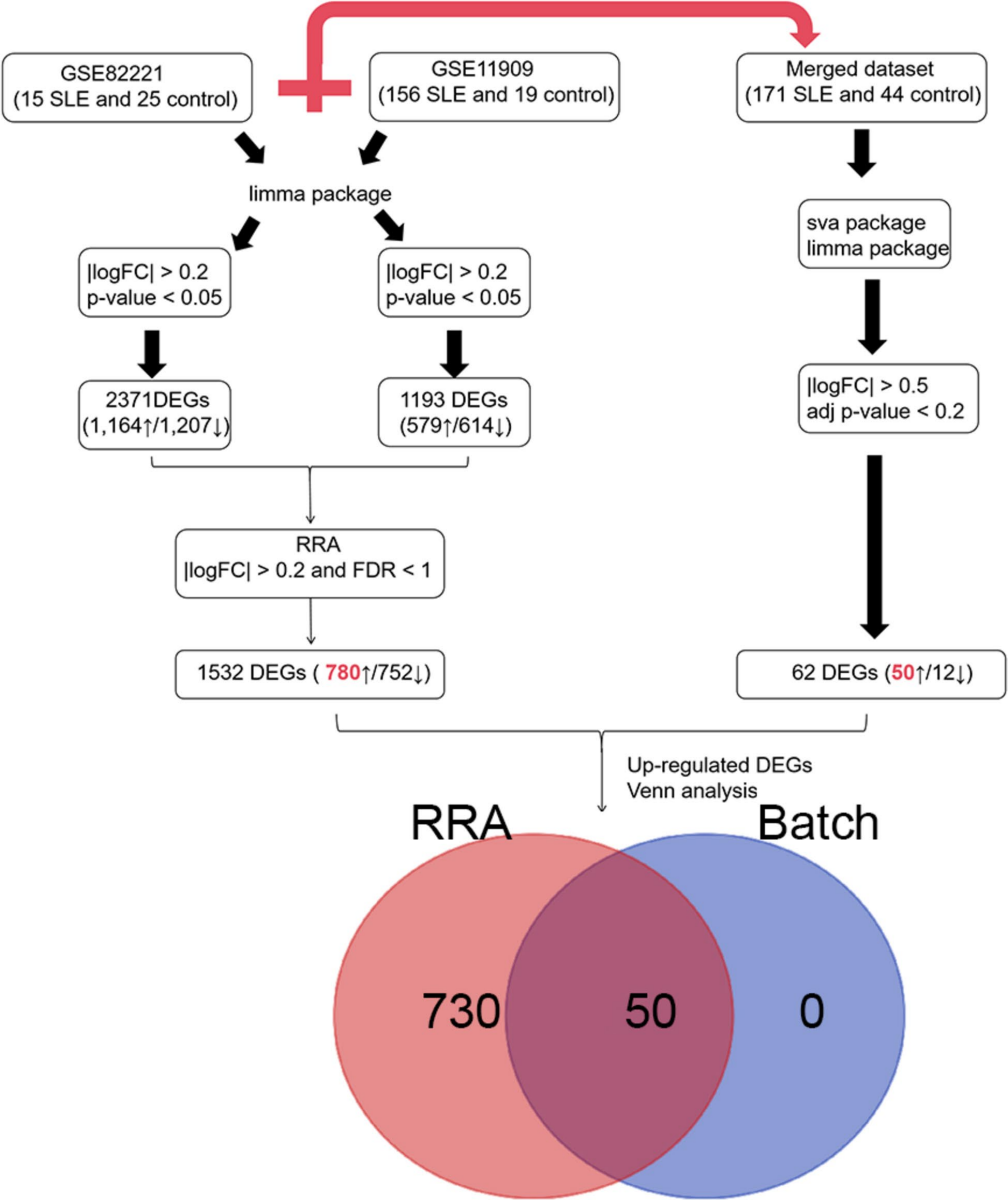
The full flow of overlapping up-regulated differentially expressed genes was identified

Based on the premise that upregulated genes may hold greater biological significance in disease pathogenesis and demonstrate superior resistance to batch effects, we prioritized upregulated genes for Venn diagram analysis (Fig. 3). Cross-dataset consistency validation (GSE82221, GSE11909, and RRA integration) revealed that 50 upregulated genes exhibited stable expression across all three analytical phases, whereas the 12 downregulated genes only met thresholds in the merged dataset phase and failed cross-platform reproducibility validation. While this focused approach enhanced result robustness, it may exclude biologically relevant downregulated targets, which are further addressed in the Discussion. This multi-stage validation strategy effectively balanced sensitivity for initial detection and specificity for consensus identification, with |log_2_FC| thresholds adapted to analytical context: 0.2 for inclusive primary screening versus 0.5 for confirmatory merged analysis.

### GO and KEGG analyses for 50 intersecting genes

**Fig. 4:**
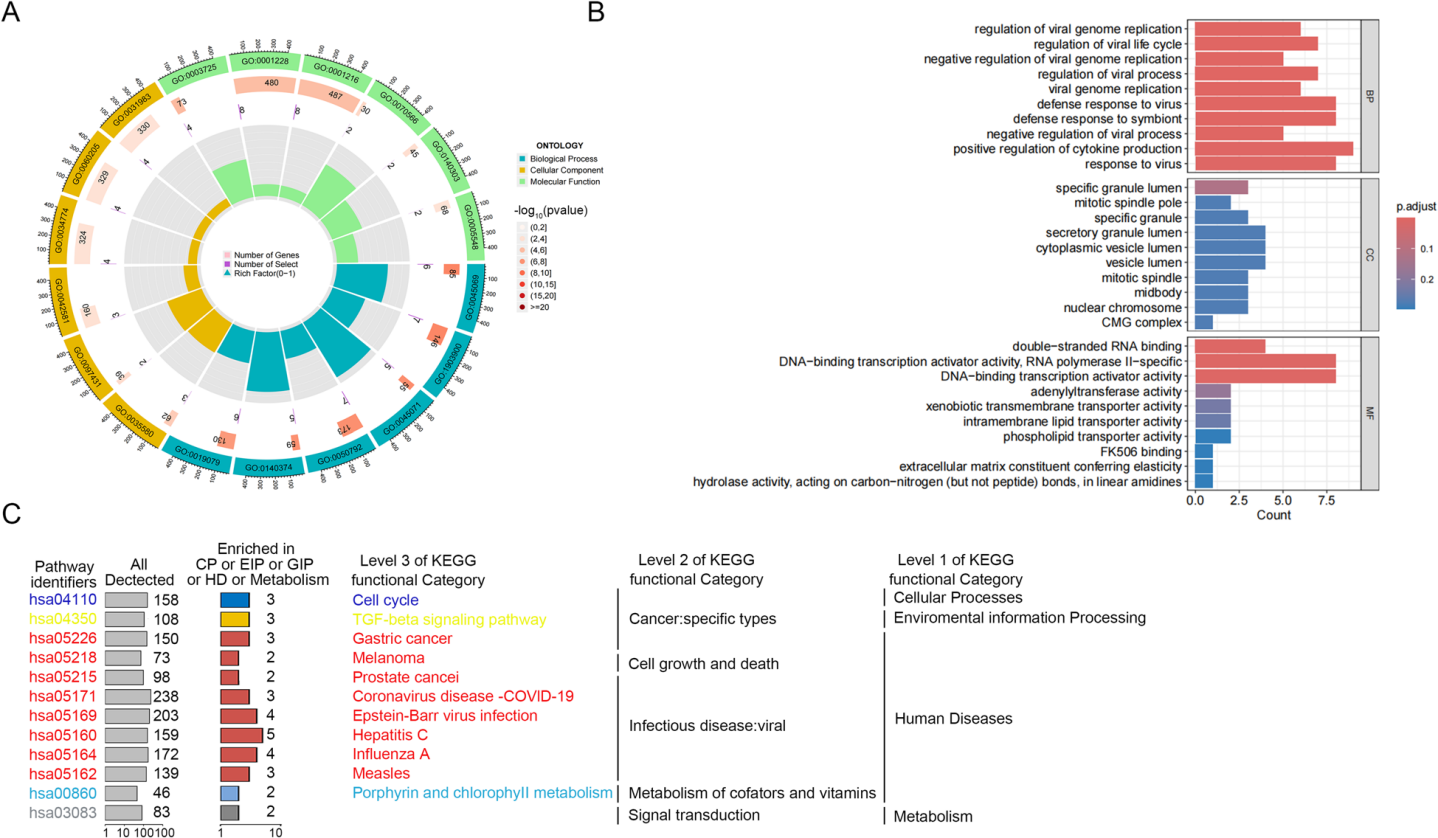
GO and KEGG analysis for 50 intersecting genes. **A** and **B** GO analysis of 50 DEGs. **C** KEGG analysis for 50 intersecting genes (https://www.kegg.jp/pathway/hsa04110; https://www.kegg.jp/pathway/hsa04350; https://www.kegg.jp/pathway/hsa05226; https://www.kegg.jp/pathway/hsa05218; https://www.kegg.jp/pathway/hsa05215; https://www.kegg.jp/pathway/hsa05171; https://www.kegg.jp/pathway/hsa05169; https://www.kegg.jp/pathway/hsa05160; https://www.kegg.jp/pathway/hsa05164; https://www.kegg.jp/pathway/hsa05162; https://www.kegg.jp/pathway/hsa00860; https://www.kegg.jp/pathway/hsa03083)

The Gene Ontology (GO) functional enrichment analysis results are categorized into three major classes: Biological Process (BP), Cellular Component (CC), and ​​Molecular Function (MF) (Fig. 4A). BP terms (blue) are predominantly enriched in GO:0019079, GO:0140374, GO:0050792, GO:0045071, GO:1,903,900, and GO:0045069. CC terms (orange) are primarily associated with GO:0035580, GO:0097431, GO:0042581, GO:0034774, GO:0060205, and GO:0031983. MF terms (green) highlight significant enrichment in GO:0003725, GO:0001228, GO:0070566, GO:0140303, and GO:0005548.

The identified BP are centered around host-virus interactions, emphasizing both viral replication regulation and antiviral defense mechanisms. Terms such as "regulation of viral genome replication," "antiviral innate immune response," and "defense response to virus" highlight genes involved in detecting viral components and activating immune pathways to suppress viral replication. Simultaneously, processes like "negative regulation of viral process" and "response to virus" suggest a dynamic interplay where the host may either restrict viral spread through innate immune signaling or encounter viral strategies that manipulate cellular machinery. This category underscores genes potentially linked to viral lifecycle control, immune evasion, and host–pathogen conflict resolution (Fig. 4B). The CC point to subcellular structures and vesicle-associated compartments critical for immune function and cell division. Terms such as "specific granule lumen," "secretory granule lumen," and "cytoplasmic vesicle lumen" imply roles in storing or transporting immune mediators during antiviral responses. Structures like the "mitotic spindle pole," "midbody," and "nuclear chromosome" may reflect viral interference with host cell division or exploitation of mitotic machinery for replication. The "CMG complex," a key helicase in DNA replication, suggests potential interactions between viral replication and host genome maintenance. Together, these components highlight spatial and structural contexts for antiviral activity, viral propagation, or host cell cycle disruption. The MF focus on ​biochemical activities tied to nucleic acid interactions, immune regulation, and membrane dynamics. "Double-stranded RNA binding" and "DNA-binding transcription activator activity" likely represent host sensors or transcription factors activated during antiviral responses. Enzymatic terms like "adenylyltransferase activity" and "FK506 binding" may involve post-translational modifications or immunosuppressive pathways. Transport-related functions ("phospholipid transporter activity") could link to membrane remodeling during viral entry/exit or immune signaling. Additionally, "extracellular matrix constituent conferring elasticity" might indicate roles in maintaining tissue integrity during infection. This category bridges molecular mechanisms to broader antiviral strategies, from viral component recognition to immune modulation.

The analysis of KEGG revealed significant enrichment of pathways encompassing cellular regulation(cell cycle [hsa04110] and TGF-beta signaling [hsa04350]), cancer-specific mechanisms including gastric cancer [hsa05226], melanoma [hsa05218], and prostate cancer [hsa05215], viral infection responses such as Epstein-Barr virus [hsa05169], hepatitis C [hsa05160], and measles [hsa05162], alongside COVID-19-related pathways [hsa05171] and metabolic processeslike porphyrin metabolism [hsa0860] and cofactor/vitamin metabolism [hsa03083]. These findings collectively highlight critical biological themes spanning cell proliferation, immune dysregulation, oncogenesis, viral pathogenesis, and metabolic adaptation.

### SLE-related gene subtypes based on consensus cluster analysis

**Fig. 5:**
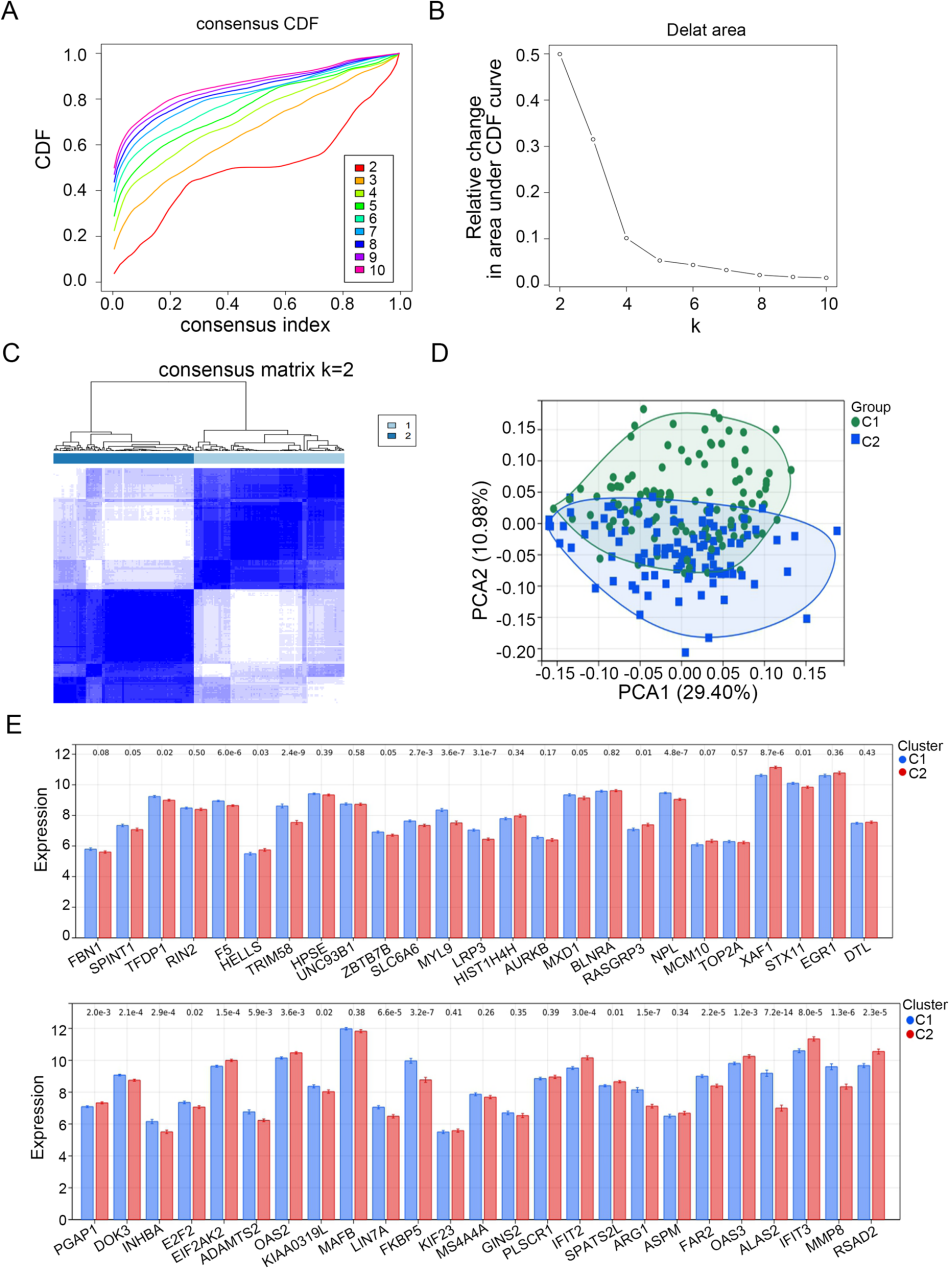
Consensus clustering analysis of the merged dataset. **A** and **B** Consensus among clusters for each category number k. **C** 215 specimens in the merged dataset were grouped into two clusters according to the consensus clustering matrix (k = 2). **D** PCA depicted the distribution for clusters. **E** The expression of 50 genes in the C1 and C2. C1, cluster 1; C2, cluster 2

In the merged dataset, genomic stratification of 215 specimens based on 50-gene expression profiles was achieved through consensus clustering methodology to delineate SLE-associated molecular subtypes. Analytical evaluation via cluster heatmap visualization and cumulative distribution function (CDF) trajectory quantification revealed optimal intergroup distinction at k = 2 clusters, evidenced by minimal incremental changes in CDF slope magnitude and maximal cluster stability (Fig. 5A-C). Principal component analysis (PCA) validation confirmed the clustering algorithm's capacity to effectively segregate specimens into two phenotypically distinct subgroups within multivariate space (Fig. 5D). Comparative expression profiling between clusters C1 and C2 demonstrated significant divergence across all 50 differentially expressed genes (DEGs), as illustrated in the hierarchical heatmap (Fig. 5E).

**Fig. 6:**
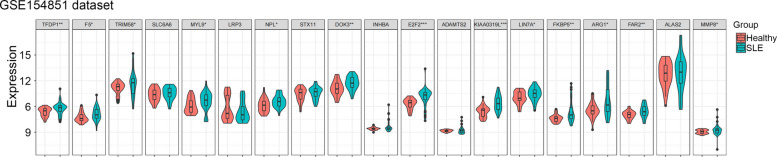
The expression of 19 DEGs in the GSE154851 dataset. SLE, Systemic Lupus Erythematosus

The fourfold diagnostic table results (Table 1) demonstrated that in the C1 subtype, 92 cases were SLE patients (83% of this subtype) and 19 were healthy controls (17%). This indicates a significant enrichment of the C1 gene expression profile in the SLE population, suggesting its potential association with disease pathogenesis. In the C2 subtype, 79 cases were SLE patients (76%) and 25 were healthy controls (24%). Although SLE patients remained predominant in C2, the higher proportion of healthy controls compared to C1 (24% vs. 17%) implies that C2 may represent a compensatory or near-healthy molecular phenotype. Collectively, the highly expressed genes in the C1 subtype are more likely to reflect core molecular features underlying SLE pathogenesis. We found that 19 genes were highly expressed in C1, including TFDP1, F5, TRIM58, SLC6A6, MYL9, LRP3, NPL, STX11, DOK3, INHBA, E2F2, ADAMTS2, KIAA0319L, LIN7A, FKBP5, ARG1, FAR2, ALAS2, and MMP8 (Fig. 5E; *P* < 0.05). The GSE154851 dataset was used as an independent dataset for expression validation. The expression levels of the 19 genes are shown in Fig. 6. As discovered, 13 genes were highly expressed in SLE, including TFDP1, F5, TRIM58, MYL9, NPL, DOK3, E2F2, KIAA0319L, LIN7A, FKBP5, ARG1, FAR2, and MMP8 (*P* < 0.05).

**Table 1:** Fourfold table diagnostic test for gene expression to predict risk for patients with SLE

		Real	
		SLE	Healthy
Predict	C1	92	19
	C2	79	25

C1, cluster 1; C2, cluster 2; SLE, Systemic Lupus Erythematosus

### Identification of hub genes with diagnostic value and development of a diagnostic model for the SLE-related module via machine learning

**Fig. 7:**
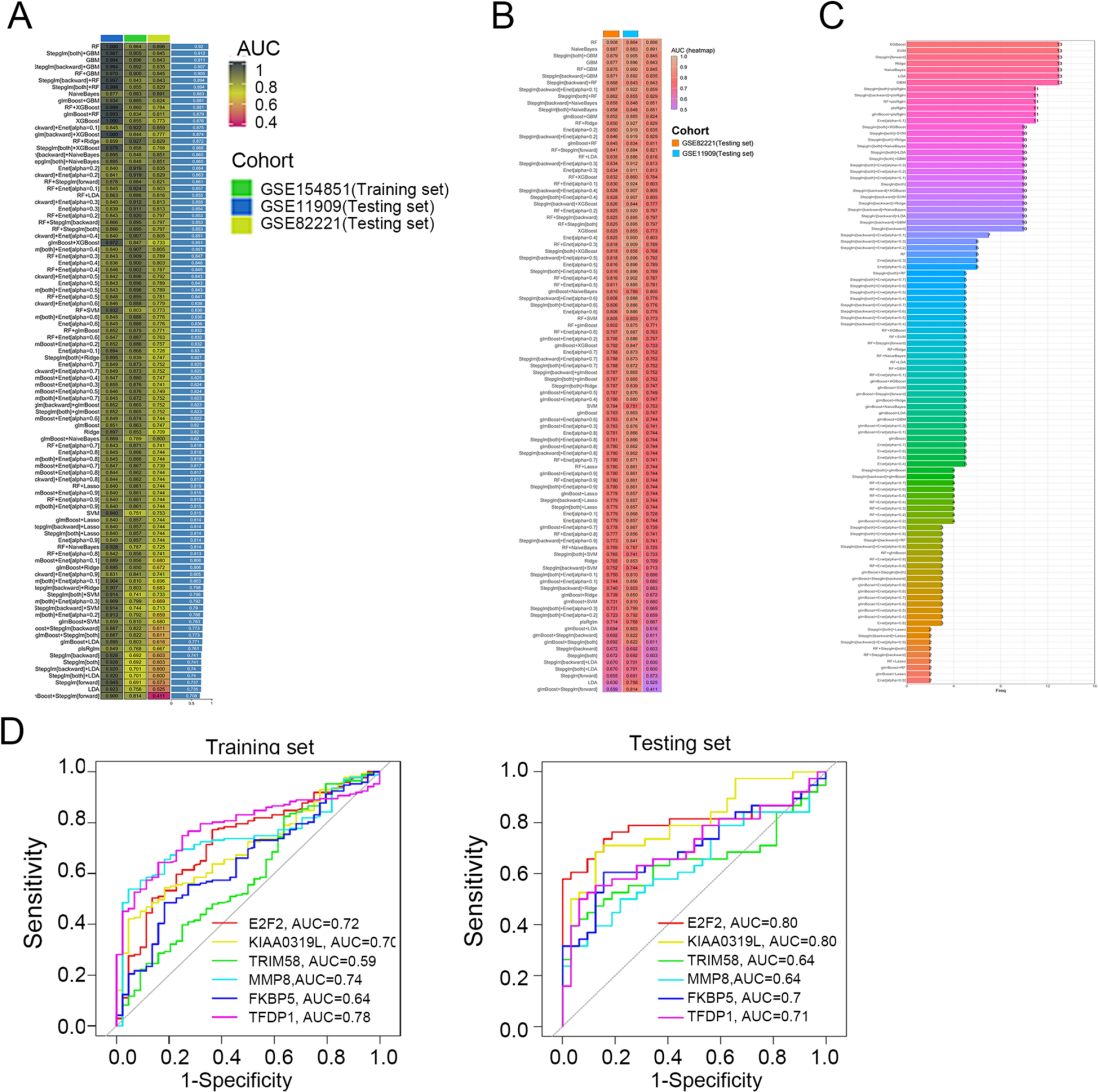
Diagnostic performance of our model. **A**-**C** 113 machine learning algorithm combinations evaluated via tenfold cross-validation. **D** ROC curves of the training cohort (GSE154851) and testing cohort (GSE82221/GSE11909)

A total of 12 machine learning algorithms were combined 113 combinations via 10-fold cross-validation to identify the most robust diagnostic model based on the 19 candidate genes. The Random Forest (RF) algorithm emerged as the optimal diagnostic model. Based on the comprehensive scores of the training set (GSE154851) and the test set (GSE82221/GSE11909), the random forest model exhibited remarkable performance. The area under the curve (AUC) reached 0.92, the accuracy rate was 88.6%, the sensitivity was 91.6%, the specificity was 85.9%, and the F1—score was 0.87 (Fig. 7A). Moreover, according to the scores of the test set, the random forest model also showed excellent performance, with the areas under the curve (AUC) being 0.908 and 0.864 respectively (Fig. 7B), establishing it as the most robust model among all evaluated approaches (Fig. 7C). RF identified six pivotal diagnostic genes—E2F2, KIAA0319L, TRIM58, MMP8, FKBP5, and TFDP1 (Fig. 7D). In the second-ranked Stepglm[both] + GBM model (test set AUC = 0.89, training set AUC = 0.85), 5 of the 10 selected genes overlapped with the RF model (E2F2, KIAA0319L, MMP8, TRIM58, FKBP5). In the third-ranked GBM model (test set AUC = 0.87, training set AUC = 0.82), all 6 RF-selected genes were included among the 13 identified genes. We evaluated the receiver operating characteristic (ROC) curves of six biomarkers (E2F2, KIAA0319L, TRIM58, MMP8, FKBP5, and TFDP1) in the training set and test set (Fig. 7D) to evaluate their ability to distinguish between classes. In the training set, TFDP1 had the highest area under the curve (AUC) of 0.78, followed by MMP8 (AUC = 0.74) and E2F2 (AUC = 0.72), while TRIM58 showed the lowest performance (AUC = 0.59). In the test set, E2F2 maintained strong discrimination with the highest AUC of 0.80, and KIAA0319L matched this AUC (0.80) after improvement. The performance of TFDP1 was slightly decreased with an AUC of 0.71, while the performance of MMP8 was significantly decreased (AUC = 0.64). TRIM58 remained the weakest biomarker in both datasets (test AUC = 0.64). The results show that E2F2 and TFDP1 exhibit relatively stable diagnostic potential across different datasets.

### Immune Landscape of the GSE154851 dataset

**Fig. 8:**
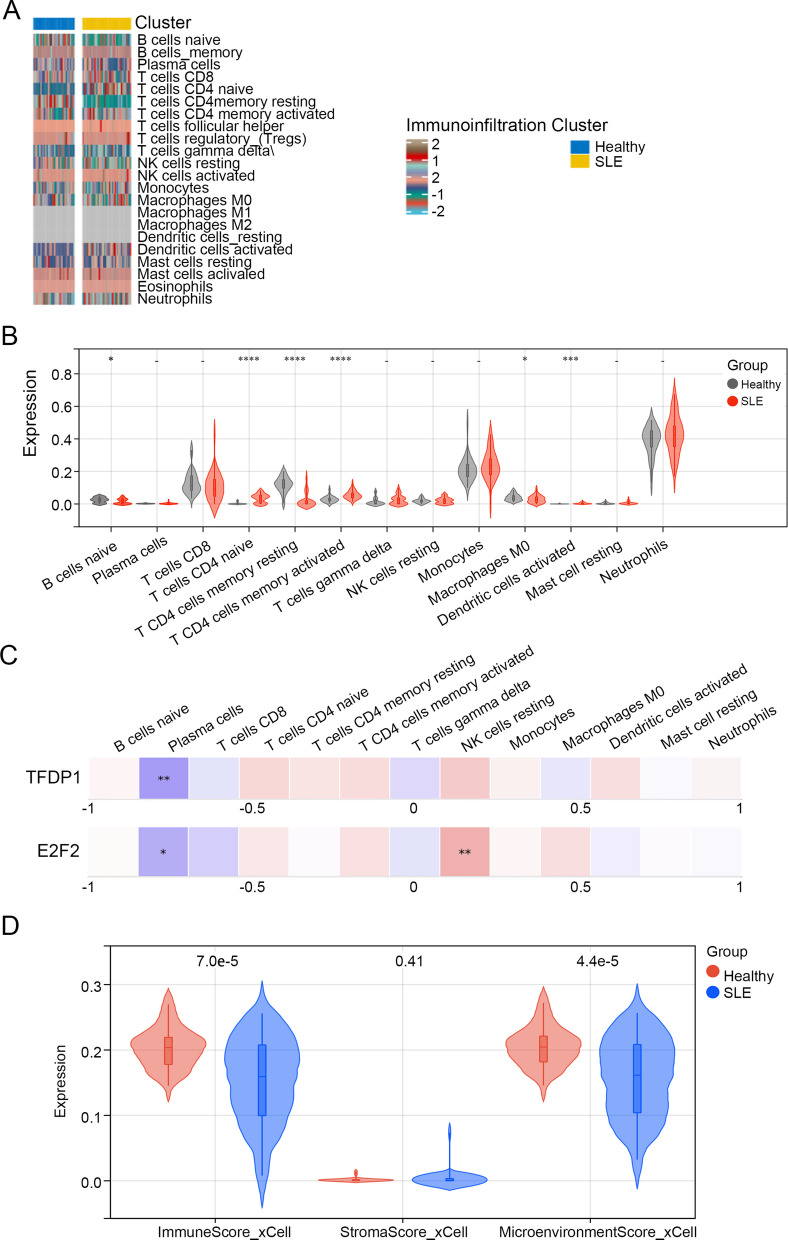
Estimation of infiltrating immune cell types in the GSE154851 dataset via CIBERSORT and xCell score. **A** Stacked barplots show the relative composition of 22 immune cell subsets in GSE154851 dataset. **B** The violin plots show the immune infiltration-related cell level in two groups. **C** The correlation between TFDP1 or E2F2 expression and immune infiltration-related cell level. **D** Violin plots comparing immune, stromal, and microenvironment scores (calculated via xCell) between Healthy (red, *n* = [X]) and SLE (blue, *n* = [Y]) groups. * *p*-value < 0.05, ** *p*-value < 0.01, *** *p*-value < 0.001, **** *p*-value < 0.0001

Several immune-related gene sets were enriched in the SLE group compared with the healthy control group, particularly concerning the innate immune system. The immune landscape in the GSE154851 dataset was then investigated utilizing the CIBERSORT algorithm. The abundances of 22 distinct immune cell types are illustrated through the stacked bar plots (Fig. 8A). Cell types not present in at least half of the samples were excluded from analysis, and their relative proportions were depicted in boxplots. The results indicated that SLE tissues exhibited higher infiltration levels of naive CD4 T cells and activated memory CD4 T cells alongside a lower infiltration level of M0 macrophages within the GSE154851 dataset (Fig. 8B; *P* < 0.05). Furthermore, there was a significant positive correlation between the expression level of TFDP1 or E2F2 and the number of plasma cells (Fig. 8C). As demonstrated in Fig. 8D, the significantly elevated immune score (SLE vs. Healthy: *p* = 7.0 × 10^–5^) indicates pervasive immune cell infiltration (e.g., plasma cells, activated CD4^+^T cells) in SLE patients, aligning with prior CIBERSORT findings of adaptive immune activation. The immune scores derived from xCell cross-validated these CIBERSORT results. The microenvironment score showed a marked divergence (SLE vs. Healthy: *p* = 4.4 × 10^–5^), confirming immune components as the primary drivers of microenvironmental alterations, whereas stromal components exhibited no statistical significance (*p* = 0.41). These findings suggest that an inflammatory microenvironment alters both the proportion and distribution of immune cells.

### E2F2 and TFDP1 have a good diagnostic performance for SLE

**Fig. 9:**
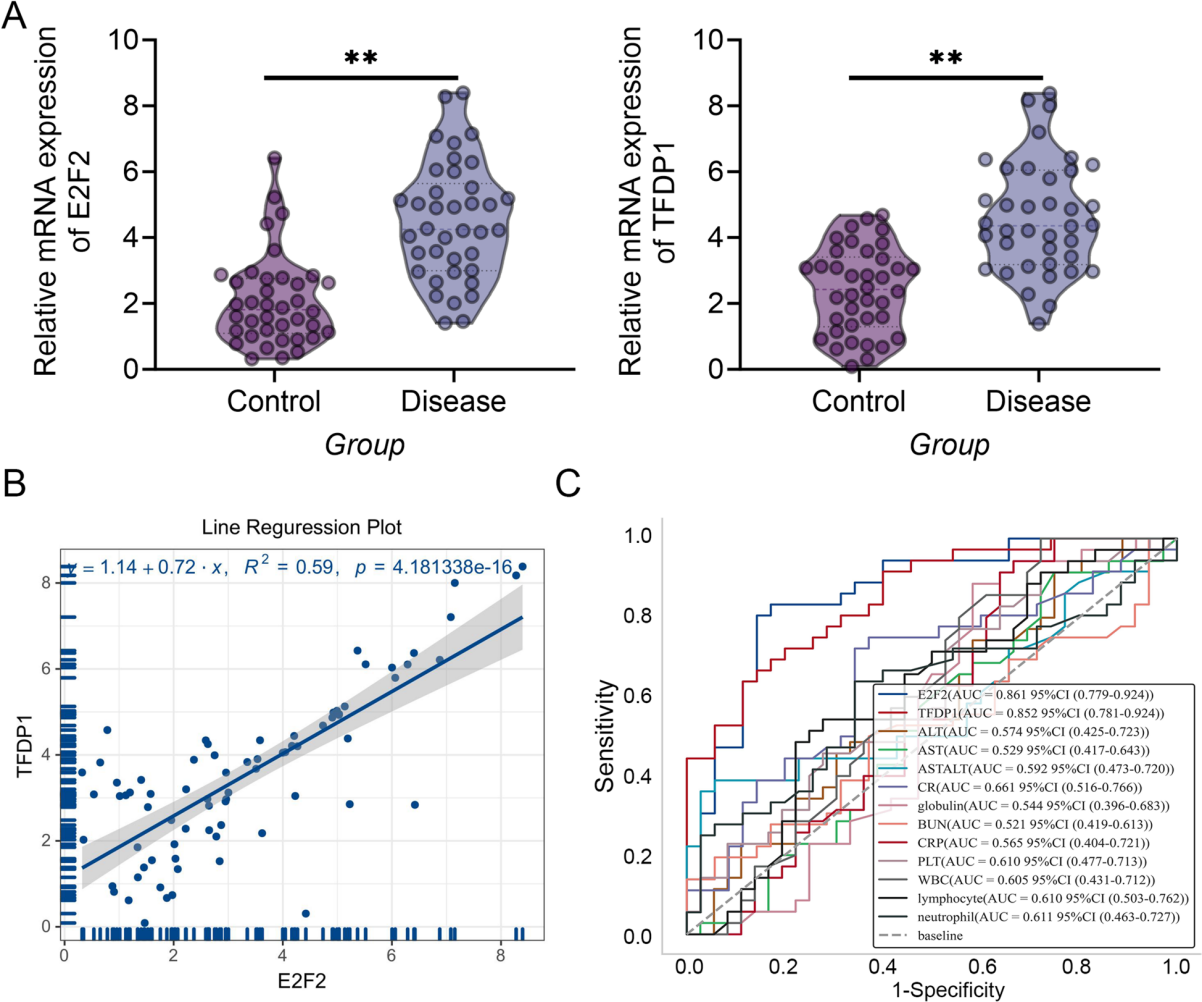
E2F2 and TFDP1 have a good diagnostic performance for SLE. **A** RT-qPCR analysis of the mRNA level of E2F2 and TFDP1 between control and disease groups. **B** Correlation analysis of E2F2 and TFDP1 expression. **C** ROC analysis of E2F2, TFDP1, gender, age, ALT, AST, AST/ALT, CR, globulin, BUN, CRP, PLT, WBC, lymphocyte (%), and neutrophil (%) in SLE. * *p*-value < 0.05, ** *p*-value < 0.01

**Fig. 10:**
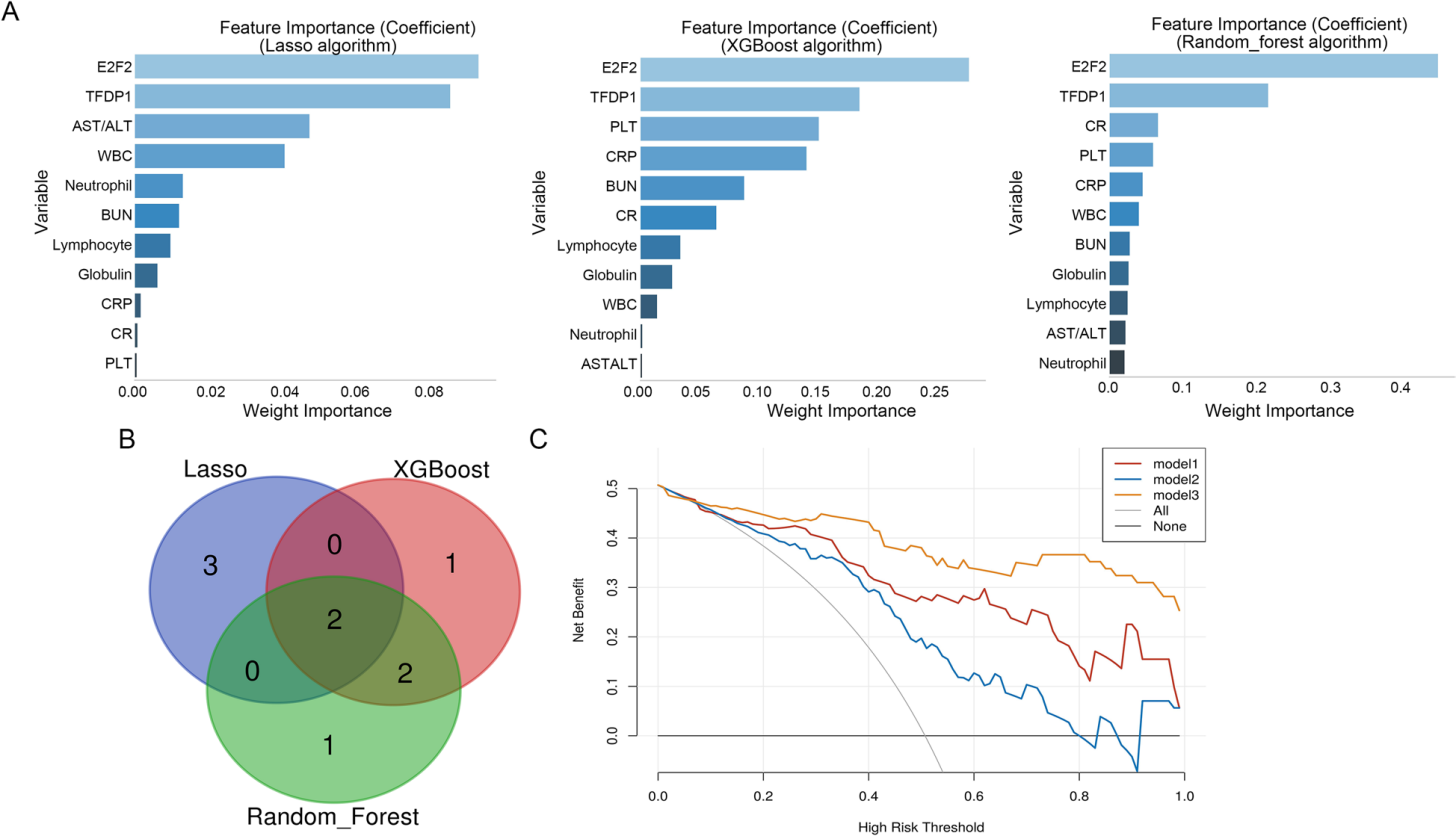
Identification of the important variables. **A** Ranking of the importance of impact factors of 15 variables based on Lasso, XGBoost, and random forest algorithms. **B** Venn analysis was used to obtain overlapped variables. **C** DCA analysis of diagnostic model of SLE, model 1 consisting of E2F2 and TFDP1; model 2 consisting of gender, age, ALT, AST, AST/ALT, CR, globulin, BUN, CRP, PLT, WBC, lymphocyte (%), and neutrophil (%); model 3 consisting of gender, age, ALT, AST, AST/ALT, CR, globulin, BUN, CRP, PLT, WBC, lymphocyte (%), neutrophil (%), E2F2 and TFDP1

Blood samples were collected from 38 non-SLE controls and 38 SLE patients. The clinical sample data included gender, age, alanine aminotransferase (ALT), aspartate aminotransferase (AST), AST/ALT, creatinine (CR), globulin, blood urea nitrogen (BUN), C-reactive protein (CRP), platelet (PLT), white blood cell (WBC), lymphocyte (%), and neutrophil (%) (Table 2). RT-qPCR results showed that the expression of E2F2 and TFDP1 was significantly up-regulated in SLE patients (Fig. 9A). Moreover, there was a significant positive correlation between E2F2 and TFDP1 expression (Fig. 9B). The diagnostic performance of E2F2 and TFDP1 was superior to that of other clinical indicators, and their AUC values exceeded 0.85 (Fig. 9C). Next, the Lasso, XGBoost, and RF algorithms were employed to assess the impact of the 15 variables (gender, age, ALT, AST, AST/ALT, CR, globulin, BUN, CRP, PLT, WBC, lymphocyte, and neutrophil, E2F2 and TFDP1) on the diagnosis of SLE, and finally the top 11 variables were selected (Fig. 10A). Subsequently, Venn analysis was conducted to identify the overlapping variables. E2F2 and TFDP1 were the overlapping genes (Fig. 10B). DCA showed the positive net benefit using Model 3 consisting of 15 variables (Fig. 10C). The diagnostic performance of Model 3/or 1 including E2F2 and TFDP1 was better than that of Model 2 without E2F2 and TFDP1. Therefore, E2F2 and TFDP1 enhanced the diagnostic performance for SLE.

**Table 2:** Clinical sample characteristics

Characteristics	non-SLE (*N* = 38)	SLE (*N* = 38)	Total (*N* = 76)
Gender			
Female	1(1.32%)	2(2.63%)	3(3.95%)
Male	37(48.68%)	36(47.37%)	73(96.05%)
Age			
Mean ± SD	51.05 ± 15.23	47.87 ± 17.12	49.46 ± 16.17
Median[min–max]	51.00[17.00,85.00]	48.00[15.00,84.00]	50.00[15.00,85.00]
ALT			
Mean ± SD	20.98 ± 11.19	29.91 ± 70.77	25.44 ± 50.53
Median[min–max]	17.10[8.30,47.60]	15.40[6.50,448.00]	16.55[6.50,448.00]
AST			
Mean ± SD	20.71 ± 7.59	25.02 ± 29.02	22.89 ± 21.30
Median[min–max]	18.50[11.00,49.90]	17.50[11.00,192.30]	18.30[11.00,192.30]
AST/ALT			
Mean ± SD	1.14 ± 0.35	1.33 ± 0.63	1.24 ± 0.52
Median[min–max]	1.23[0.57,1.86]	1.22[0.42,3.21]	1.23[0.42,3.21]
CR			
Mean ± SD	54.25 ± 9.41	86.93 ± 146.48	70.59 ± 104.40
Median[min–max]	53.35[37.70,77.40]	58.10[35.10,947.80]	56.30[35.10,947.80]
Globulin			
Mean ± SD	28.97 ± 3.56	30.56 ± 9.27	29.76 ± 7.02
Median[min–max]	28.30[21.90,38.90]	28.45[20.40,71.60]	28.30[20.40,71.60]
BUN			
Mean ± SD	4.80 ± 1.54	5.75 ± 3.47	5.27 ± 2.71
Median[min–max]	4.54[2.11,9.11]	4.83[2.72,18.16]	4.58[2.11,18.16]
CRP			
Mean ± SD	5.36 ± 8.53	11.47 ± 27.20	8.41 ± 20.25
Median[min–max]	2.15[0.20,32.87]	1.82[0.01,115.18]	1.93[0.01,115.18]
PLT			
Mean ± SD	224.76 ± 95.98	190.37 ± 76.46	207.57 ± 87.91
Median[min–max]	205.00[81.00,578.00]	184.00[68.00,387.00]	193.00[68.00,578.00]
WBC			
Mean ± SD	6.94 ± 2.04	6.60 ± 2.89	6.77 ± 2.49
Median[min–max]	6.85[3.00,11.90]	6.15[2.60,14.20]	6.40[2.60,14.20]
Lymphocyte (%)			
Mean ± SD	23.86 ± 8.64	20.59 ± 9.53	22.23 ± 9.19
Median[min–max]	27.10[7.80,42.50]	20.00[7.60,42.50]	22.30[7.60,42.50]
Neutrophil (%)			
Mean ± SD	66.50 ± 9.57	70.56 ± 11.74	68.53 ± 10.83
Median[min–max]	64.90[46.70,87.70]	69.85[45.70,88.90]	68.65[45.70,88.90]

*WBC* White blood cells, *PLT* Platelet, *CR* Creatinine, *BUN* Blood urea nitrogen, *CRP* C-reactive protein, *AST* Aspartate Aminotransferase, *ALT* Alanine Transaminase

## Discussion

SLE is a chronic diffuse connective tissue disease caused by the abnormal activation of the immune system, which attacks its tissues [6]. Therefore, in this study, SLE-related datasets were analyzed for immune infiltration, and the results indicated that SLE tissues exhibited higher infiltration levels of naive CD4 T cells and activated memory CD4 T cells alongside a lower infiltration level of M0 macrophages. SLE is a systemic disease involving multiple systems, with the most intuitive manifestation being the impact on the skin [13]. The skin is the largest organ in the human body. Establishing immune memory in the skin is an important component of the adaptive immune response [14]. Once naive T cells are activated by antigen-presenting cells, a small fraction of them will differentiate into precursor memory T cells [15]. Tissue-resident memory T cells are formed by the stimulation of naive T cells and reside in peripheral tissues to mediate the rapid immune response to similar stimuli [16]. Although this immune response helps protect the body against local infection, the prolonged residence of tissue-resident memory T cells in the skin in autoimmune-related skin diseases such as vitiligo can lead to recurrent disease flare [15].

Macrophages, the key components of the innate immune system, are involved in regulating the initiation, progression, and resolution of numerous inflammatory diseases [17]. Multiple studies have suggested a causal relationship between the presence or activation of macrophage cells and the development of autoimmune diseases. Macrophages are the first line of defense against pathogen invasion, which can dissolve, engulf and destroy the pathogens [18, 19]. In conclusion, the results of immune infiltration analysis obtained from our dataset were consistent with previous reports, indicating that this dataset is of research value.

Based on the significant biological relevance of upregulated genes in disease pathogenesis and their superior resistance to batch effects, integrated bioinformatics analyses, machine learning models, experimental validation, and clinical ROC modeling (AUC for E2F2 testing set = 0.80; AUC for TFDP1 training set = 0.78) identified E2F2 and TFDP1 as potential diagnostic biomarkers for SLE, enhancing result robustness. However, this strategy may have overlooked potential downregulated targets like ZFR2, MYBL1, RAVER2, PID1, FGF9, USP53, S1PR5, and AK5​​ (not previously reported in SLE), and literature-reported SLE-associated biomarkers including ABCB1 [20], PLEKHA1 [21], BCL11B [22], and the lupus nephritis-specific gene PRSS23 [23]. These exclusions likely occurred because some (e.g., PRSS23) may specifically correlate with SLE subtypes (e.g., lupus nephritis), while the study prioritized pan-SLE diagnostic biomarkers, and downregulated genes exhibit greater susceptibility to batch effects, leading to insufficient stability in cross-dataset validation. In contrast, E2F2 and TFDP1 demonstrated superior cross-platform consistency and analytical stability (AUC > 0.7 in both training and testing sets), supported by experimental validation, indicating resilience to biological noise and validating their selection as diagnostic targets. Compared to conventional serological markers (e.g., C3/C4) that fluctuate with disease activity, E2F2/TFDP1exhibit unique transcriptional stability in PBMCs, showing consistent upregulation across cohorts. As heterodimeric transcription factors, E2F2-TFDP1 may be involved in the regulation of plasma cell differentiation—a critical driver of autoantibody production in SLE [34] and their overexpression correlates with elevated plasma cell infiltration, suggesting they may serve as functional indicators of disease activity. While downregulated genes like ZFR2​and MYBL1 may hold biological relevance, their susceptibility to technical batch effects and inconsistent validation outcomes limits diagnostic translatability. Conversely, the E2F2-TFDP1 axis satisfies critical biomarker criteria for SLE: potential mechanistic relevance to SLE pathophysiology (particularly plasma cell dysregulation), robust analytical stability across platforms and datasets (maintaining AUC > 0.7), and clinical feasibility for early detection.

As a member of the E2F family of transcription factors, E2F2 functions by forming heterodimers with dimerization partner (DP) proteins, such as TFDP1, through its dimerization domain. This interaction enables nuclear localization and sequence-specific DNA binding via the DNA-binding domain (DBD), thereby regulating transcription of target genes involved in DNA replication and synthesis [24]. The E2F2-TFDP1 heterodimer has been reported to modulate cell cycle progression by controlling G1-S and G2-M phase transitions [25] and may contribute to abnormal lymphocyte proliferation [26]. During plasma cell differentiation, the E2F2-TFDP1 axis may be activated in the B-cell-to-plasma-cell transition, exhibiting a positive correlation with PRDM1 (Blimp-1), the master regulator of plasma cell differentiation. Mechanistically, this axis has been suggested to cooperate with the chromatin remodeler BRG1 (SMARCA4) to epigenetically open the XBP1 locus [27–29]. In inflammation regulation, the E2F2-TFDP1 complex has been suggested to modulate the transcriptional activity of NF-κB pathway genes (e.g., IL-6 and TNF-α), potentially limiting excessive inflammatory responses [30, 31]. These findings suggest a potential dual regulatory role in maintaining immune homeostasis. Collectively, these mechanisms suggest that the E2F2-TFDP1 axis may play a role in immune dysregulation in SLE.

E2F2, a transcription factor, belongs to the E2F gene family [32–34]. It is regulated by cyclin-dependent kinase (CDK), and CDK inhibitors regulate and activate cyclins A and E to promote cell cycle progression. In addition, E2F2 has a nuclear localization signal near the cyclin A-binding domain, suggesting that E2F2 can enter the nucleus and thus regulate its own activity in a cell cycle-dependent manner. Many studies have demonstrated that E2F2 is highly expressed in a variety of tumors, such as ovarian cancer, breast cancer, gastric cancer, and liver cancer, which is closely related to the occurrence and development of tumors [33, 35]. E2F2 expression is closely related to cancer immune infiltration, however, E2F2 is largely unknown in SLE.

The transcription factor dimerization ligand (TFDP) family, in the normal physiological state, can form a dimer with the E2F family, which plays an important role not only in the regulation of cell cycle, cell differentiation, cell apoptosis and other important functions, but also in the occurrence and progression of cancer [24, 36]. Currently, the TFDP family includes three members, TFDP1, TFDP2, and TFDP3. TFDP1, first discovered in 1993 [37], is highly expressed in various human tissues and has a sequence similar to E2F1. Under normal circumstances, TFDP1 binds to E2F1 as a heterodimer that later binds to E2F transcription sites. Meanwhile, TFDP1 binds to E2F1, and the interaction between TFDP1 and pRB, another key cell cycle regulator, is essential [38]. Similar to E2F2, the role of TFDP1 in SLE remains largely unclear [31].

Our study illustrated a significant positive correlation between the expression level of TFDP1 or E2F2 and the number of plasma cells. Plasma cells have a key function in the pathogenesis of SLE [39]. To be specific, they can secrete antibodies for a long time, produce protective memory, and participate in the long-term immunity. In SLE, autoantibodies can be continuously produced, while the excessive production of autoantibodies leads to inflammatory cascades and large-scale immune responses, eventually causing organ damage and disease recurrence [40]. TFDP1 and E2F2 may regulate cell proliferation and cell cycle, and thus potentially promote plasma cell proliferation to accelerate the occurrence and development of SLE.

In addition, ALT, AST, AST/ALT, CR, globulin, BUN, CRP, PLT, WBC, lymphocyte, and neutrophil are the diagnostic markers of SLE [41, 42]. In this study, these markers were combined with sex, age, and the expression levels of E2F2 and TFDP1 to develop a diagnostic model. Our findings indicated that the diagnostic performance of the model incorporating E2F2 and TFDP1 was significantly superior to that of the model excluding these two genes. In future work, widely developed deep learning techniques and their applications to more complex scenarios could be further explored [43].

## Limitations

Several limitations of this study should be acknowledged. First, the clinical validation cohort was relatively small (*n* = 76) and derived from a single center, which may limit the generalizability of the findings. Second, the publicly available GEO datasets lacked consistent clinical annotations (e.g., age, sex, disease activity scores, and treatment status), precluding adjustment for potential confounders in the bioinformatics analyses. Third, our study focused on upregulated genes based on their superior cross-platform reproducibility, which may have excluded biologically relevant downregulated targets. Fourth, although the Random Forest model demonstrated strong diagnostic performance, the high training set AUC warrants caution regarding potential overfitting, and further validation in larger, multi-center, prospective cohorts is needed. Finally, functional validation experiments (e.g., knockdown/overexpression studies) were not performed; future studies should investigate the mechanistic roles of E2F2 and TFDP1 in SLE pathogenesis to confirm their potential as therapeutic targets.

In conclusion, our study identified E2F2 and TFDP1 as potential diagnostic biomarkers for SLE through integrated bioinformatics analysis, machine learning modeling, and clinical RT-qPCR validation. Both genes were significantly upregulated in SLE patients and positively correlated with plasma cell infiltration. Incorporation of E2F2 and TFDP1 into clinical diagnostic models improved discriminatory performance. These findings warrant further validation in larger, independent cohorts and functional studies to elucidate the mechanistic roles of these biomarkers in SLE pathogenesis..

## Data Availability

The datasets used and/or analysed during the current study are available from the corresponding author on reasonable request. The datasets generated and/or analysed during the current study are available in the Gene Expression Omnibus (GEO) repository (GSE82221, GSE11909, and GSE154851). The datasets generated and/or analysed during the current study are available in the Gene Expression Omnibus (GEO) repository (GSE82221, GSE11909, and GSE154851).

## Abbreviations

SLE: Systemic lupus erythematosus
PBMCs: Peripheral blood mononuclear cells
GEO: Gene Expression Omnibus
RRA: Robust rank aggregation
DEGs: Differentially expressed genes
GO: Gene Ontology
KEGG: Kyoto Encyclopedia of Genes and Genomes
ROC: Receiver operating characteristic
DCA: Decision curve analysis
ALT: Alanine aminotransferase
AST: Aspartate aminotransferase
CR: Creatinine
BUN: Globulin, blood urea nitrogen
CRP: C-reactive protein
PLT: Platelet
WBC: White blood cell
RT-qPCR: Reverse transcription-quantitative polymerase chain reaction
AUC: Area under the curve
IFN-I: Type I interferon
DREAM: Dimerization partner, RB-like, E2F and multi-vulval class B
